# In Memoriam: J. Richard Baringer, MD (1935–2025)

**DOI:** 10.1002/ana.78295

**Published:** 2026-07-14

**Authors:** John E. Greenlee, John W. Rose, Kathleen B. Digre, Mark B. Bromberg

**Affiliations:** ^1^ Department of Neurology University of Utah School of Medicine Salt Lake City UT USA; ^2^ Department of Ophthalmology University of Utah School of Medicine Salt Lake City UT USA

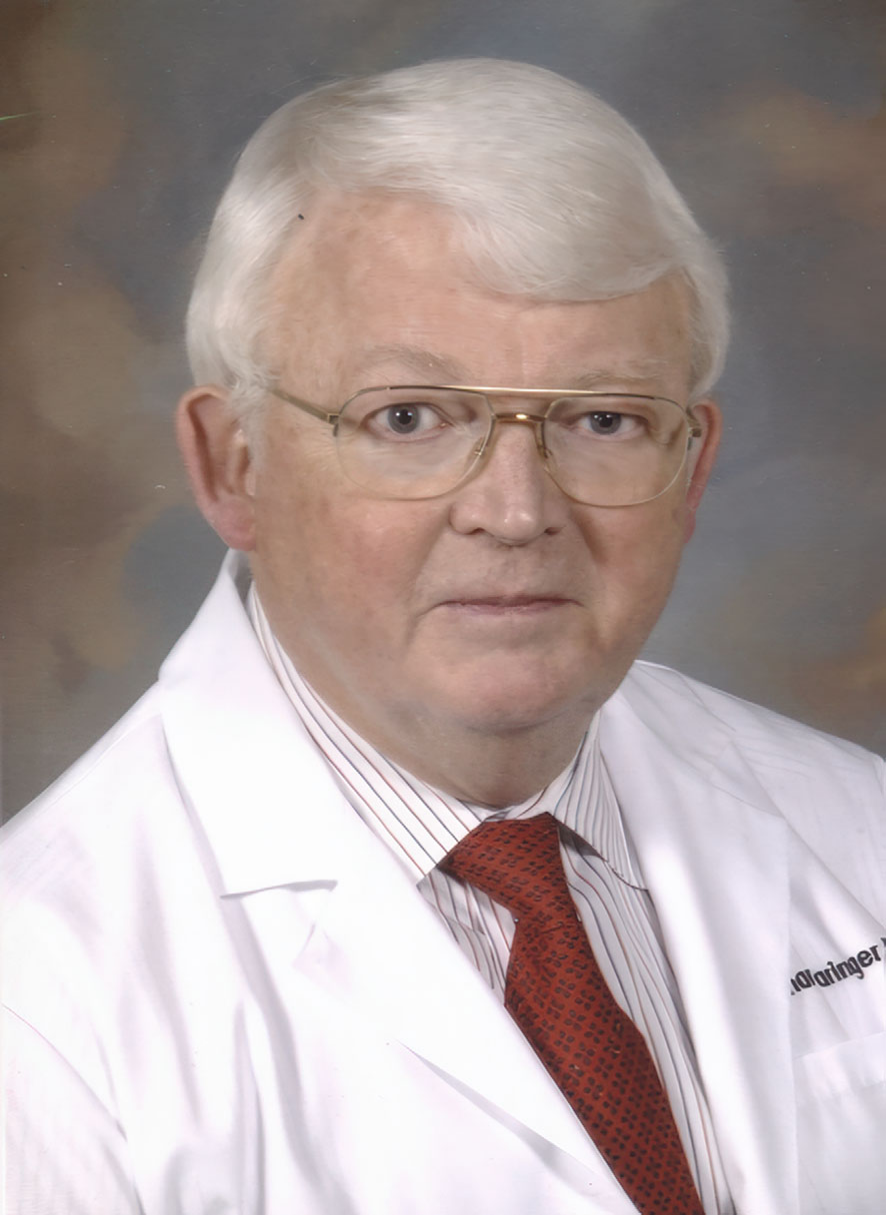

John Richard Baringer: January 19, 1935 to June 22, 2026.

Dr John Richard (Dick) Baringer died peacefully on July 22, 2025, bringing to a close a long and extraordinarily productive life within and beyond the field of neurology. Dick had celebrated a joyous 90th birthday on January 19, 2025, but then declined rapidly in health over the next several months. In his death, the world lost an outstanding clinician–scientist and human being.

Dick was born in Columbus, Ohio, on January 19, 1935. By age 10 years, at an age when many boys are dreaming of a life as a major professional athlete (or possibly a dreaded pirate), he knew that he wished to become a physician. He received his BS from the Ohio State University in 1955, and his MD from Case Western Reserve University in 1959. Dick trained at Massachusetts General Hospital from 1959 to 1968, first as Intern, Resident, and Fellow in Neurology and Resident in Pathology, and subsequently as Resident in Neurology and Fellow in Neuropathology. He became Assistant in Neurology and Instructor in Neuropathology in 1968.

In 1969, Dick moved to the University of California, San Francisco, as Assistant Chief of the Neurology Service at the Veterans Administration Hospital, San Francisco and as Assistant Professor in Residence, and then Assistant Professor in the Department of Neurology (1960–1973), progressing to Professor in Residence of Neurology and Pathology (1976–1982) and Chief of Neurology at the San Francisco Veterans Administration Hospital (1974–1982). From 1978 to 1979 Dick served as Acting Chair of Neurology at University of California, San Francisco.

While still in Boston, Dick had begun work on the neuropathology of herpes simplex virus encephalitis. Following his move to San Francisco, working with Kenneth Johnson, Stanley Prusiner, Jerry Wolinsky, and many other individuals, he built one of the world's premier programs in central nervous system infections and neurovirology.

In 1982, Dick was recruited to become Chair of the Department of Neurology at the University of Utah School of Medicine, assuming leadership of a department with a faculty of only 10 neurologists, limited national presence, and a tenuous residency program. On July 1, his first day as Chair, his secretary greeted him with: “Dr Baringer, we have a problem: one of the residents who was to join our program this morning has just resigned after being admitted to veterinary school.” Dick took this in his stride, recruited faculty carefully, made the program attractive to aspiring residents, and built strong research efforts in multiple areas, including neuroinfectious disease, neuroimmunology/multiple sclerosis, and neurogenetics. Over his tenure as Chair, he developed a nationally prominent department of over 50 individuals.

Academic medicine is often described as a three‐legged stool, with the legs comprising patient care, teaching, and research. Dick excelled in each of these areas. He was a superb clinician across the broad field of neurology. He was an effective and supportive teacher of both medical students and residents. He was a major figure in the field of neurovirology, where he carried out ground‐breaking studies in herpes simplex persistence and latency, including the role of the virus in Mollaret's meningitis.^1^, ^2^, ^3^ He was involved in pivotal clinical trials in the treatment of herpes simplex encephalitis^4^; published original research in areas ranging from central nervous system toxoplasmosis to measles, rubella, and prion diseases^5^, ^6^, ^7^, ^8^; and, late in his career, demonstrated persistence of herpes simplex DNA in human brains.^9^ As Chair, he strongly advocated for women in neurology. He was an effective recruiter of both faculty and residents, and an invaluable mentor, in particular for junior faculty and trainees. He later said: “My role was to bring people in, give them as much support and encouragement as I could, and let them do their thing.”

What we term the “three‐legged stool,” however, also has a fourth very important leg, comprising what one gives to the world as a person and how one's life affects others. Dick's personal academic accomplishments within medicine and neurology were major, and he was a highly effective chair. He was active nationally in the American Academy of Neurology, the American Neurological Association, and the American Association of Neuropathologists, serving on the editorial board of *Neurology* from 1977 to 1982, and the *Journal of Neuropathology and Experimental Neurology* from 1983 to 1985. He held numerous positions within the American Neurological Association, including member and then Chair of the Long Term Planning Committee (1978–1980), member and then Chair of the Membership Committee (1983–1985), Secretary (1993–1997), President Elect (1998–2000), and President (2000–2002). From 1991 to 1993, he served as President of the American Neurological Association's Society for Experimental Neuropathology. He was active in the National Multiple Sclerosis Society, both locally and nationally.

In addition to his career as a neurologist, Dick was a man of many interests and accomplishments. While still in medical school, he was exposed to classical music, and this remained a lifetime love, leading him to serve on the board of the Utah Symphony and to promote a “Healthcare Night” for physicians at the Utah Symphony. He also developed a program whereby medical students could attend concerts and have the same exposure to classical music that he had experienced. Beyond this, Dick was a skilled skier and fly fisherman, as well as an avid squash ball player. He greatly enjoyed, each year, going on trekking expeditions with his wife, Jeannette, in the US and places abroad, including Nepal. He was an outstanding photographer, qualifying for and taking courses with Ansel Adams; and he enjoyed the challenge of producing outstanding film and digital images, sending prints as holiday gifts to his family and friends.

Most of all, Dick was a wonderful leader, colleague, and friend, with an extraordinary and unselfish ability to draw individuals to him. He maintained a broad interest in the field of neurology, continuing to attend departmental activities well into retirement, and serving as a source of advice and counsel to department members, as well as many others.

Dick is survived by his wife and long‐time collaborator in research, Dr Jeannette Townsend, and by his sons, William, John, and Gregory. He is greatly missed by all those whose lives he touched.

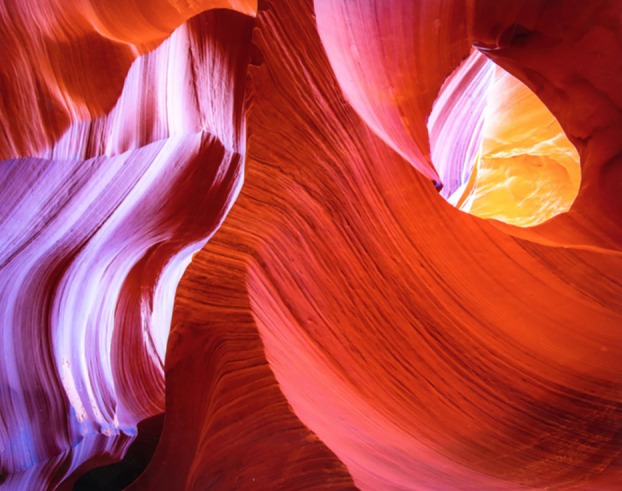

J. Richard Baringer: Sandstone slot canyon detail: Lower Antelope Canyon, Page, Arizona, 2003.
